# Using live-action 360-degree video to assess the impact of exposure duration on eyewitness identification accuracy at high confidence in children and adults

**DOI:** 10.1186/s41235-025-00692-9

**Published:** 2025-12-17

**Authors:** Kara N. Moore, Dara U. Zwemer, James Michael Lampinen, Pia Pennekamp, Thomas J. Nyman, Pekka Santtila, Julia Korkman, Jan Antfolk, Chenxin Yu

**Affiliations:** 1 University of Utah, Salt Lake City, USA; 2 University of Arkansas, Fayetteville, USA; 3 University of Reading, Reading, UK; 4 New York University Shanghai, Shanghai, China; 5 Abo Akademi University, Turku, Finland

## Abstract

The pristine conditions hypothesis postulates that highly confident witnesses will be highly accurate, even when witnessing conditions are poor. Recent research has extended this to children and concluded that, on average, child-eyewitnesses who are highly confident are rather accurate (i.e., 85–97%, Winsor et al., Journal of Experimental Psychology. General 150:2387–2407, 2021). However, this has only been tested in good witnessing conditions. Since then, research in adults has shown that, in some poor witnessing conditions, the high confidence-accuracy relationship breaks down. We sought to determine if highly confident child and adult eyewitnesses would be highly accurate even in poor witnessing conditions. We presented 1,055 participants (485 young children, 357 older children, and 213 adults) with a 360-degree live-action mock-crime video in a virtual reality headset. To test whether witnessing conditions impact children’s confidence-accuracy relationship, we manipulated exposure duration (short-6 s, long-34 s) at encoding and the presence of the culprit in the lineup identification task. Surprisingly, memory strength was weak for all age groups under good and poor witnessing conditions. There were so few high confidence identifications in adults that the confidence-accuracy relationship could not be plotted. Importantly, we found that the pristine conditions hypothesis does not hold regardless of the state of the witnessing condition. This research suggests that there are boundary conditions to the pristine conditions hypothesis and that further research is needed to determine the boundary conditions of the pristine conditions hypothesis.

## Public significance statement

Eyewitness identifications provide valuable evidence in criminal cases but are prone to error. Eyewitness misidentifications are a leading type of evidence in wrongful convictions. To assess eyewitness identification accuracy, researchers and practitioners have turned to eyewitness confidence as a predictor of accuracy. Researchers have found that high confidence is associated with high identification accuracy under pristine lineup administration procedures. However, people may not accurately account for the impact of witnessing conditions (e.g., lighting, distance) when they judge the accuracy of their memory. This could lead to high confidence not being associated with high accuracy. Therefore, we tested whether high confidence would indicate high accuracy in children and adults under optimal and suboptimal conditions (i.e., viewing duration) using live-action 360-degree videos to increase realism. We found that high confidence did not predict accuracy which has implications for using confidence as an indicator of eyewitness accuracy in criminal cases.

## Introduction

Eyewitness identifications are important evidence in criminal cases, yet an eyewitness’s ability to discriminate between guilty and innocent people in a lineup is error prone. Due to the need to discern eyewitness reliability, researchers have been increasingly interested in understanding how well eyewitness confidence predicts accuracy. In an influential paper, Wixted and Wells (2017) proposed a claim that Giacona et al. (2021) call “the pristine conditions hypothesis.” The claim is that when pristine conditions are used by police, highly confident suspect identifications will be “remarkably accurate” (Wells & Wixted, 2017, p. 10). Pristine conditions refer to the state of several *system variables,* which are variables that can be controlled by the police. The pristine conditions consist of a fair lineup, one suspect in a lineup, unbiased instructions, double-blind administration, and the collection of confidence immediately after the identification. Wixted and Wells (2017) theorized the strong relationship between high confidence and accuracy would hold even if estimator variables are poor (e.g., the lighting is dim, the distance is far). Estimator variables refer to variables that cannot be controlled by police. Wixted and Wells (2017) found support for the pristine conditions hypothesis, focusing on young adult eyewitnesses. However, since its publication there have been mixed findings on whether the pristine conditions hypothesis holds in adults depending on the condition of estimator variables (see Moore et al., 2024, for review).

Millions of children witness crime around the world each year. In the United States alone, over half a million children ages 12–17 years were victims of crime in 2022 (Bureau of Justice Statistics [BJS], 2023). However, Wixted and Wells (2017) did not test the pristine conditions hypothesis in children. Since then, one study has examined children’s confidence-accuracy relationship under pristine conditions, but it did not test the impact of estimator variable conditions on the confidence-accuracy relationship (Winsor et al., 2021). In the current research, we assessed the impact of estimator variable conditions on child (5–13 years old) and adult eyewitnesses' confidence and accuracy. We simulated some realistic conditions that are not typically featured in crime stimuli in research, by using live-action 360-degree videos in virtual reality headsets, to enhance the generalizability of our conclusions about the confidence-accuracy relationship in eyewitness identification.

### Lineup performance in adulthood and childhood

In a lineup, eyewitnesses are presented with a set of individuals and asked if any of them are the culprit. One of the individuals in the lineup is the suspect. The suspect may be guilty or innocent. The other individuals are called fillers. Fillers are innocent individuals who are included to provide plausible alternatives to the suspect. A lineup with a guilty suspect is called a culprit present lineup. A lineup with an innocent suspect is called a culprit absent lineup.

Children generally perform worse than adults at correctly rejecting a lineup that does not contain a culprit (Fitzgerald & Price, 2015; Parker & Carranza, 1989; Pozzulo & Balfour, 2006; Pozzulo & Lindsay, 1998). There is less certainty about the developmental differences between children and adults at correctly identifying a culprit from a culprit present lineup. Some research finds that, from age 5, children perform as well as adults at correctly identifying the culprit (Parker & Carranza, 1989; Pozzulo & Balfour, 2006; Pozzulo & Lindsay, 1998). Other research finds that children perform worse than adults at correctly identifying the culprit (Fitzgerald & Price, 2015; Keast et al., 2007). Fitzgerald and Price’s (2015) meta-analysis found that children (4–17) made fewer correct identifications than adults. This difference decreased as children’s age increased. Regarding face recognition development in general, Kinnunen et al. (2013) examined over 600 5–15-year-olds face recognition abilities and found rapid development of children’s face recognition ability up to 8–9 years, which then slowed through the age of 11 years. The existing research suggests there are developmental differences at identification accuracy.

### The confidence-accuracy relationship in adult eyewitness identification

Confidence and suspect identification accuracy are strongly related in adults in laboratory studies that use pristine conditions (Sauer et al., 2019; Wixted & Wells, 2017). Until the mid-2010s, calibration analysis (how well eyewitness’ confidence corresponds to a group of eyewitnesses’ average identification accuracy) was the leading approach for analyzing confidence-accuracy (Juslin et al., 1996). Mickes (2015) introduced confidence-accuracy characteristic curves which differ from calibration analysis in that they do not include filler identifications, allowing researchers to address the court’s question of the probability that the suspect is guilty. Wixted and Wells (2017) reanalyzed eyewitness identification studies using confidence-accuracy characteristic curves and found that adult eyewitnesses were remarkably accurate under pristine conditions. A definition of remarkably accurate is needed to assess when evidence supports the pristine conditions hypothesis. Based on the examples given by Wixted and Wells, we take the claim of remarkably accurate to refer to accuracy of 95% or higher. In reviewing confidence-accuracy studies, Wixted and Wells (2017) state, “It is visually apparent that in most cases, high confidence accuracy is very high (95%-100% correct).” (p. 30).

Wixted and Wells (2017) hypothesized that this relationship would not be affected by estimator variables. Many studies have tested this in adults. Some find that estimator variables do not impact accuracy at high confidence (Carlson et al., 2017; Semmler et al., 2018), while many others have found that poor estimator variable conditions reduce accuracy at high confidence below remarkable accuracy (Giacona et al., 2021; Grabman et al., 2019; Lin et al., 2019; Lockamyeir et al., 2020; Nyman et al., 2019, 2025; Semmler et al., 2018; Winsor et al., 2021). The exceptions found to the pristine conditions hypothesis include poor face recognition ability (Grabman et al., 2019), slow identification decisions (Grabman et al., 2019), retention intervals of 10 min (Lin et al., 2019), viewing distances of 12.5, 20 m, and 45-110 m (Lockamyeir et al., 2020; Nyman et al., 2019, 2025), the presence of multiple poor estimator variables (Giacona et al., 2021; Nyman et al., 2025), when decisions are justified by familiarity (Grabman et al., 2019), young age (i.e., 4–9 years, Winsor et al., 2021) and adulthood (i.e., 30 + years, Colloff et al., 2017).

### The confidence-accuracy relationship in  child eyewitness identification

Few studies have examined confidence-accuracy in children. Keast et al. (2007) explored children’s (10–14 years old) and adults’ confidence-accuracy calibration under pristine conditions and found that children had worse calibration than adults. Children were more overconfident than adults. In one study, the researchers used a *hypothesis disconfirmation* manipulation. In the hypothesis disconfirmation condition, they emphasized variables that could negatively affect the eyewitness' memory of the suspect to determine if it could help participants to account for negative impacts on their memory. However, children had poor calibration even in this condition. Winsor et al. (2021) collected a large sample of young age (4–6), middle age (7–9), and older age (10–17) children to test confidence-accuracy using pristine conditions and optimal estimator variable conditions. They found that, for high confidence suspect identifications, young children (87%) were less accurate than middle (91%) or older (97%) children. They concluded that “all three age groups achieved high suspect ID accuracy at high confidence” (Windsor et al., 2021, p. 2,397). However, the children in the two youngest age groups were not remarkably accurate. Neither study specified whether the pristine condition of double-blind lineup administration was used. Critically, no research has examined the impact of estimator variables on the pristine conditions hypothesis in children exclusively or as compared to other age groups. Existing research has shown that estimator variables impact children’s memory (e.g., Havard et al., 2017), but a critical question is what happens to children’s accuracy at high confidence under poor estimator variable conditions. The most systematic investigation of the impact of estimator variables on the pristine conditions hypothesis in children (5–17 years) are by Nyman et al., (2019, 2025) using live targets in live settings. Nyman et al. (2025) investigated the effects of distance, lighting, and facial masking on identification accuracy (*N* = 1,325; age range of 5–90) and found that high confidence was not associated with high accuracy under certain viewing conditions. However, the study did not independently assess the confidence–accuracy relationship in children or contrast it with that of adults. Nyman et al. (2019) investigated distances of 5–110 m in outdoor settings with optimal lighting (*N* = 1,588; age range of 6–77) and found that the confidence-accuracy relationship (high confidence 81–100%) held up to 40 m for all age groups (young children (6–11), older children (12–17), young adults (18–44) and older adults (45–77). However, exact accuracy rates among participants who were highly confident are not provided and high confidence identifications were rare beyond 40 m.

### Confidence-accuracy relationship theories

#### Constant likelihood ratio

Recognition memory is often understood through a signal detection account. Assume a simple signal detection model of the lineup task (Wixted et al., 2018). Each lineup member produces a match value to memory. For any witness, one member of the lineup will produce the highest match and that lineup member will be compared to a response criterion that determines whether the lineup is rejected or that lineup member is accepted. The theory assumes there is one response criterion for each level of confidence.

Stretch and Wixted (1998) proposed that participants adjust their response criterion following a constant likelihood ratio rule. This rule states that participants adjust the criterion in such a way that the probability that the item is old, given that it has been judged to be old, remains constant under different conditions. When memory is strong, the high confidence response criterion is less stringent than when memory is weak. This requires people to be able use metacognition to accurately monitor their memory and adjust their response criteria to a confidence scale according to their memory strength (Semmler et al., 2018; Stretch & Wixted, 1998). A complication in this account arises in that even adults often have imperfect knowledge of how witnessing conditions are likely to affect accuracy. For instance, researchers have found that laypersons' opinions of the impact of estimator variables on eyewitness memory diverge from those of experts (Benton et al., 2006). In recognition memory and eyewitness contexts, researchers have theorized that people are not perfectly accurate at adjusting their response criteria as witnessing conditions change (Semmler et al., 2018; Stretch & Wixted, 1998). More recently, Mickes and Wixted (2021) wrote, "To be sure, there is undoubtedly some low level of discriminability where the confidence­ accuracy relationship will break down." According to this account, what drives placement of the high confidence response criterion is overall memory strength, which is adjusted imperfectly as memory strength becomes very weak. However, this account has not made clear at what memory strength the confidence-accuracy relationship will break down or why. Cases where high confidence-accuracy relationships have diverged from the pristine conditions account have often involved low values of d' (i.e., weak memory strength). For instance, in Lockamyeir et al., (2020, Experiment 2) d' was only about .30 in the longest distance condition. In Giacona et al. (2021) d' was about .57 in the poor view condition. However, it remains to be determined at what levels of discriminability the confidence-accuracy relationship breaks down at various ages.

A critical question is to what extent the constant likelihood ratio theory fits eyewitnesses when conditions are suboptimal. Most research is conducted in conditions that are optimized in a variety of ways. For example, researchers make sure there are no distractions from the crime in research. However, in the real world, eyewitnesses face the choice of what to attend to. Testing this question allows us to establish if the constant likelihood ratio theory underlying the pristine conditions hypothesis holds in eyewitnesses.

### Confidence and metacognitive development

The developmental literature finds that certainty and metacognitive abilities come online early in life (Baer & Kidd, 2022; Godfrey et al., 2023) and that they develop throughout childhood (Godfrey et al., 2023). Three-to-five-year-old children were more certain when they accurately remembered an item than when they were inaccurate (Lyons & Ghetti, 2011). However, children’s ability to calibrate their confidence to a scale develops in childhood with younger children (less than 8 years old) generally being overconfident (Roebers, 2002; Roebers & Howie, 2003; Roebers et al., 2004, 2007). Ten-year-olds perform better at calibrating confidence to accuracy than 8-year-olds (Roebers, 2002; Roebers & Howie, 2003; Roebers et al., 2007). While overconfidence decreases with age, even adults have been shown to be overconfident (Dunning et al., 2003). Procedural metamemory—one’s knowledge of their own memory abilities—and declarative memory—one’s knowledge of the influence of memory strategies and task features – similarly come online during early childhood but develop throughout childhood (Godfrey et al., 2023; Liu et al., 2018). For example, 5-year-olds know that salient and unlikely events are more memorable than low salient or common events (Ghetti & Alexander, 2004). However, these abilities are still developing as children’s ability to distinguish between easy and difficult to remember stimuli develops into adulthood (Tsalas et al., 2015). This literature indicates that children have some ability to understand their own memory and calibrate their confidence judgments to their memory but that these abilities are not matched to adults. This suggests that children may not be remarkably accurate when they are highly confident in their eyewitness identification decision.

### The impact of exposure duration on lineup identification

#### Adulthood

In the current research, we manipulated the estimator variable of exposure duration to determine how the confidence-accuracy relationship in children and adults is impacted by estimator variables. The visual system samples information about visual stimuli over time, and therefore the more time one has to view an image the more detailed the representation of the image becomes (Loftus, 1985). Longer exposure durations result in higher lineup identification accuracy than shorter exposure duration (Brewer et al., 2000; Memon et al., 2003; Palmer et al., 2013). A meta-analysis of this literature found a reliable effect of exposure duration on identification accuracy (Bornstein et al., 2012; but see, Carlson et al., 2016). Most of these studies use exposure durations that vary in terms of 10 s of seconds (e.g., 12 s vs. 45 s). Memon et al. (2003) additionally found that participants in the long exposure duration condition made fewer false identifications from culprit-present and culprit-absent lineups. Regarding the confidence-accuracy relationship, Palmer et al. (2013) compared adult witnesses who viewed a target individual for either 5 s or 90 s and then made a lineup judgment from a culprit present or absent lineup. They found that shorter durations reduced the proportion of correct identifications, however, highly confident witnesses were very accurate regardless of exposure duration. Researchers have also found that longer exposure durations to actual culprits are associated with higher identifications of the suspect from a lineup than shorter exposure durations (Horry et al., 2014; Valentine et al., 2003).

#### Childhood

Only a few studies have explored the effect of exposure duration on child witnesses. Goodman and Reed (1986) found that 6-year-old children were more likely identify the culprit from a culprit present lineup than 3-year-old children (*N* = 48). Critically, they coded the duration that participants looked at the culprit during the event and found that 6-year-old children spent more time looking at the culprit than 3-year-old children. Leippe et al. (1991) found that 5–6 and 9–10-year-olds (*N* = 84) performed better at identifying a culprit they were exposed to for 6 min than one they were exposed to for 9 s. Gross and Hayne (1996) had 34 5–6-year-old children watch an event with culprits who were present for one hour or 30 s. The children were more likely to make a correct decision when they were exposed to the culprit for longer on culprit present lineups but not on culprit absent lineups. This research suggests that children’s face memory is impacted by exposure duration similarly to that of adults.

### Simulating witnessing a crime

Existing research in the eyewitness field has primarily been conducted in the laboratory. However, eyewitness memory is different in field studies than lab studies (Eisen et al., 2017; Foster et al., 1994; Nyman et al., 2020). It is vital to recreate real-world conditions to be able to draw generalizable conclusions. The use of live-action 360-degree video embedded into virtual reality headsets provides an avenue to increase realism as it can emulate real-world perceptual attributes, environmental scanning, and emotional investment for viewers. Indeed, research has demonstrated that virtual reality can produce emotional experiences similar to those experienced in real environments (Brey, 2014; Coelho et al., 2009; Wallach et al., 2009). For example, military applications have shown that virtual reality can simulate the emotional stress of combat (Malta et al., 2020). Furthermore, participants that view eyewitness videos in virtual reality make more errors on lineups compared to participants who watched the video in 2D, suggesting that 2D presentation may not fully map onto real-world eyewitness events (Nyman et al., 2020). Critically, the use of live-action 360-degree video in virtual reality headsets enables us to simulate several variables of witnessing a crime that may not be featured in 2D stimuli.

### Current research

Participants viewed a live-action 360-degree video of a minor theft in a virtual reality (VR) headset that featured two culprits. After viewing the video, participants completed two lineups, one for each culprit. Mansour et al. (2017) examined the impact of administering multiple lineups to adults and found little to no impact of identification decisions and confidence. Participants each viewed one culprit absent and one culprit present lineup, with culprit presence counterbalanced by culprit. We anticipated that age and exposure duration would impact the confidence-accuracy relationship and identification decisions. Identification decision predictions were pre-registered at OSF prior to data analysis [https://osf.io/p2jm9]. Confidence-accuracy relationship predictions were developed before data analysis but were inadvertently not pre-registered.

1. *Confidence-accuracy relationship.* In the long duration condition, high confidence accuracy will not differ between age groups. In the brief duration condition, high confidence accuracy will be higher for adults than for either of the child groups.
2. *Identification accuracy*. Identification accuracy, as measured by d’ and by ROC pAUC, will be lower in the brief exposure condition than the longer exposure condition. Identification accuracy, as measured by d’ and by ROC pAUC, will be lower for younger children than for older children and lower for older children than for adults.
3. *Hits (correct identifications of culprit).* Hits will be higher in adults than children on culprit present lineups. Hits will be higher in older than younger children. Hits will increase with age. Hits will be higher in the longer exposure duration than the shorter exposure duration.
4. *Correct Rejections (rejections of absent lineups).* Correct rejections will be higher in adults than in children. Correct rejections will be higher in older children than younger children. Correct rejections will increase with age. Correct rejections will be higher in the long exposure duration than the short exposure duration.
5. *Choosing Rates (identifying anyone from a lineup).* Choosing rates will be higher in children than adults. Choosing rates will be higher in older children than adults. Choosing rates will decrease with age. Choosing rates will be lower in the long exposure duration than the short exposure duration condition in both culprit-present and culprit-absent lineups.^1^

### Method

#### Transparency and openness

We report how we determined our sample size, all data exclusions (if any), all manipulations, and all measures in the study, and the study follows JARS (Appelbaum et al., 2018).

### Design

The study was approved by the educational institutions’ ethics boards. We used a 2 (exposure duration: short (6 s), x long (34 s) × 2 (culprit presence: present or absent) mixed design with culprit presence as a within participants variable. Exposure duration was randomized. We used six-person simultaneous lineups and developmentally appropriate confidence scales. We report all data exclusions, manipulations, and measures in the study, except for physiological measurements, eye tracking, heart rate, and perspiration. None of which have been analyzed yet. These variables are excluded from the current manuscript because they are still being processed.

### Participants

We planned a stopping rule of 30–40 high confidence identifications in each between-subjects condition. However, an interim check of sample size revealed that this number was infeasible to reach without many years of data collection. As a result, we chose to discontinue the data collection early. This early analysis revealed that memory strength was quite poor. This insight alone is worth considering given that we simulated several factors that are typically ignored in most eyewitness identification experiments.

The final sample of this study included 1055 participants with 485 younger children (i.e., 5–8 years; *Mdn* = 7.25), 357 older children (i.e., 9–13 years; *Mdn* = 10.50), and 213 adults (i.e., 18–35 years; *Mdn* = 20.00). Participants had to be five years of age or older and to have no medical conditions that could hinder them from or cause them harm from viewing VR content (i.e., photosensitive epilepsy or seizures, blindness or low vision not corrected with glasses or contacts, deafness or low hearing that could not be corrected with hearing assistance, cognitive impairment, dementia, pregnancy, or heart conditions). Participants who did not complete the study or provide their date of birth were excluded. Participants self-identified as 63.10% White, 10.90% Multiracial, 9.90% Hispanic or Latin, 5.50% Black or African American, 4.80% Asian, 2.80% as American Indian or Alaska Native, 0.40% Native Hawaiian or Pacific Islander, and 2.60% self-described. As for gender, participants self-identified as 52.00% female, 46.50% male, 1.30% self-described, and 0.70% chose not to answer. Of the participants, 525 participants viewed the long duration video, and 530 participants viewed the short duration video. As for location, 201 participants were tested in a lab in the Western United States, 562 at a children’s museum in the Southwestern United States, and 282 at children’s museums in the Western United States.

### Materials

#### VR headset

The virtual reality videos were presented in Varjo Aero virtual reality goggles. These goggles provide some of the highest video resolution of any VR goggles on the market at 35 pixels per degree (PPD) with a 115-degree field of view, and they contain state-of-the-art eye-tracking technology at a frequency of 200 Hz. The goggles automatically adjusted to the pupillary distance of each participant (i.e., the distance between a participant’s pupils) and provided real-time data about changes in pupil size as an index of autonomic arousal (Wang et al., 2018).

#### Virtual reality videos

*Kitchen Demonstration Video* The kitchen demonstration video was played for approximately one minute and featured different elements of a kitchen appearing and slowly changing colors, as if designing the space (Ylinen, 2022). The 360-degree video was developed by Varjo, and was used to immerse participants in the VR experience and ensure that they would not become motion sick during the crime video.

*Mock-Crime Video* The mock-crime video was filmed from a static location with an Insta360 Titan 360-degree camera with up to 11 k resolution. To help the reader understand our materials, 2D versions of the videos and a screenshot of a portion of the crime are available on OSF [https://osf.io/63e2v/]. However, it is important to note that these materials do not provide an exact representation of the view distance or resolution of the videos because they are flattened versions of the videos with artifacts. One would need to watch the video using the same headsets and program that we used in the study to see a true representation of the video. We used top-of-the-line cameras, headsets, and computers, but they still limited us to playing the video in 4k resolution. It is important to note that resolution is not the same in 360-degree video as it is in 2D video. It is approximated that to compare 360-degree video resolution to 2D video resolution one must divide the resolution by 4. Therefore, the 2D equivalent of the video resolution was 1080p. An additional complication to measuring video resolution is that the video was displayed in a headset from a computer while the computer also performed eye tracking. Therefore, while the video resolution was objectively at 4k the video may have appeared in a consistently worse resolution in the headset. Unfortunately, there is no way to measure this, but our lineup data suggest that participants were capable of identifying the culprits from the video.

Children viewed the scene from a seated position, and they were able to look 360 degrees around them in the video. The video was filmed from the 1st person perspective such that viewers had the perspective of being an onlooker. The video was set in a park with actors playing catch and jogging in the background. In the video, a woman named Ambrosia greets the participant before showing a friend items that she purchased. She sits down on a bench with the bags on either side of herself. Two men appear and approach her on each side. After speaking to Ambrosia, each man grabs one of her bags and runs in opposite directions. Then, another man who was sitting near Ambrosia talks to her and the participant, stating the participant could help Ambrosia figure out who stole her bags and instructing that they will remove the VR goggles and answer questions.

Two versions of the video were created to manipulate exposure duration. In the short video, the culprits were featured for 6 s, and the total video length was 3 min. In the long video, the culprits were featured for 34 s, and the total video length was 5 min. In the longer video, Ambrosia showed her friend more items from her bag and talked to the culprits for longer about one of her purchases (i.e., a pickle jar) before they stole the bags than in the short video.

#### Lineups

We created six-person simultaneous culprit present and culprit absent lineups for each culprit that featured a red “X” as the not present option. There was a single version of each culprit absent lineup that featured six innocent fillers. There were six versions of the culprit present lineups in which five of the innocent fillers were included alongside the culprit, each excluding a different filler. This allowed each filler from the culprit absent lineups to be included as fillers in the culprit present lineups in every possible configuration. The positions of the individuals were randomized in the lineups by participant. The lineups featured photographs of faces cropped from their shirt collars upwards with a solid white background.

*Filler Selection* We used a description task to generate descriptions of the culprits to use to select fillers. In the description task, 18 volunteers described each culprit. Any feature mentioned by at least half of the raters was used to generate a description. The description provided for Culprit 1 was “White male, blue eyes, light brown hair, early 20 s, thin face.” The description provided for Culprit 2 was “White male, light brown hair, early 20 s, round face.” To enable blind raters to select fillers, the authors selected a pool of mugshot-style photographs that matched each culprit by appearance from various face databases. A new set of volunteers (*N* = 33) were presented with 12 mugshots (11 fillers and the culprit) for each culprit. They were asked to rate how well each face matches the description from *Not at all* = 0 to *Absolutely* = 100. We selected six fillers that received average ratings of 57.39–75.85% for Culprit 1, and 64.18–72.54% for Culprit 2 to include in the lineups.

We then conducted a mock-witness task (approved by the 3rd authors’ human ethics board) in which CloudResearch-approved Amazon Mechanical workers *(N* = 666, usable *n* = 663) were asked to choose from lineups based on the witness' descriptions. The position of the perpetrator and the presence of the culprit were randomized. Each participant made decisions on five lineups, including two lineups used in the present study and 3 lineups used for other studies. Participants were asked to make a lineup decision based on the witness’ description of the suspect (e.g., a witness described a suspect as follows: "White male, blue eyes, light brown hair, early 20 s, thin face." Which suspect best matches this description?). The culprit-present lineup for Culprit 1 had an average Tredoux *e* of 5.65, and the culprit-absent lineup for Culprit 1 had a Tredoux* e* of 4.14. The culprit-present lineup for Culprit 2 had an average Tredoux *e* of 4.14, and the culprit-absent lineup for Culprit 2 had a Tredoux *e* of 5.09.

#### Confidence scale

*Development* Bruer et al. (2017) found that probabilistic scales (e.g., “Rate your confidence from 0–100%”) may exacerbate social influences on children’s decision-making criteria, unlike categorical scales. This result may relate to metacognitive abilities that are underdeveloped in children that help adults to consider decision-making criterion and confidence (Bryce & Whitebread, 2012; Roebers, 2002). We developed a 4-point, categorical confidence scale that included the options very unsure, unsure, sure, and very sure with corresponding emojis. For the development of the scale, we conducted a pilot study including 18 child participants. The total sample of children included seven females, 10 males, and one individual who did not provide their sex. The average age of the included children was 8.24. Children were asked what emotion they thought six different emojis displayed. Then, all possible combinations of emojis were paired, and children were asked which emoji they thought displayed a more sure expression. Finally, children were asked to order the emojis from most to least sure. We selected and ordered four emojis for the confidence scale based on children’s responses.

*Training.* We utilized three example questions to train participants on the confidence scale before it was used (Roebers, 2002; F. Buehler & C. Roebers, personal communication, October 21, 2022). The high confidence question was, “What is your name?” The low confidence question was, “What do you think my best friend’s name is?” The middle confidence question was, “How old am I (the research assistant who posed the question)?”.

### Procedure

Child participants and some of the adults participants were recruited via exhibits at partnering museums.  At the museum locations, three park benches were set up in a u-shape in front of a table containing the VR equipment. Additionally, some adult participants were tested in the laboratory at a computer table. The research assistants working with participants never  saw the videos to enable double administration.

At the museum locations, the research assistant completed the consent process with the adult participant or guardian using a Qualtrics survey on a tablet. The guardian provided demographic information for the child. Afterward, children were escorted to a bench and were assented with a tablet. The research assistant described the Shimmer device and asked for the child’s permission before putting it on. The research assistant asked for permission to put the VR headset and headphones on the participant. The participant then watched the kitchen demonstration video. Afterward, the research assistant checked if the participant was motion sick. If so, the study was discontinued. Participants were not aware that they would witness a crime. Before the video was played, on-screen and verbal instructions indicated that the participant was about to meet a woman named Ambrosia and provided some background information about her. They were then asked to watch what happened. After the video ended, the research assistant asked for permission before taking the VR headset, headphones, and Shimmer device off. Then, the research assistant walked them through a post-task survey.

The post-task Qualtrics survey was administered on a tablet. Participants were asked questions to assess motion sickness first. If the participants felt unwell, the study was discontinued. Each participant was given an example culprit present lineup and an example culprit absent lineup. Participants were given unbiased lineup instructions that indicated that they would be asked to determine if a specific object was present, but that it may or may not truly be present. The instructions indicated that the participant should select the large red “X” if the object was not present. The culprit present lineup contained vehicles and participants were asked if they could identify a car from the lineup. If the participant chose correctly, the research assistant read scripted feedback to confirm that it was the car. If the participant chose incorrectly, the research assistant read scripted feedback to explain where the car was before prompting them to try again. The culprit absent lineup contained foods, and participants were asked if they could identify an apple from the lineup. If the participant was correct, the research assistant confirmed that “X” was the correct choice. If the participant was wrong, the research assistant indicated that they should have chosen the red “X,” and participants were prompted to try again. After the example lineups, children were given instructions about and received three example questions to teach them how to use the confidence scale. Children were prompted with scripted feedback and allowed to try to answer each question until they answered correctly.

After the examples, participants were given instructions for the first culprit lineup. Participants were told that they would see six pictures of people’s faces, and that they may or may not see one of the two men who took Ambrosia’s bags. They were asked to click the face of the person who stole the bags if they saw him or, if they did not see him, to choose the red X. Research assistants oriented the tablet away from themselves and toward the participants during the lineup to enable the double-blind lineup administration. After responding to the lineup, participants gave a confidence rating on the emoji confidence scale. Then, the process was repeated for the second lineup. Each participant saw one culprit present and one culprit absent lineup. Which lineup was present and absent and which culprit’s lineup was presented first was randomly assigned and counterbalanced across participants. No feedback was given about participants’ responses to the lineup or the confidence scale.

Participants were asked to tell the researcher anything else they wished to about the study and about the video quality. Then, participants in the Southwestern United States and adults in the lab in the Western United States were debriefed as per the institution’s institutional review board. Participants at the Western United States children’s museums were not debriefed, as instructed by the institution’s institutional review board. Participants were then escorted out of the exhibit or lab and all equipment was cleaned.

## Results

Behavioral results are shown in Tables 1, 2 and 3. Data are available on OSF [https://osf.io/63e2v/]. Of the 1,056 participants, 655 people (approximately 62% of the sample) had used virtual reality goggles previously. As can be seen, across conditions, performance was relatively poor. Correct rejections of culprit absent lineups ranged from 41 to 53%. Suspect identifications in culprit present lineups ranged from 12 to 25%. We also calculated d’ for each condition (Mickes et al., 2014), which ranged from .14 in the short duration younger child witness condition to .66 in the long duration adult witness condition. Diagnosticity, measured as the proportion of correct suspect identifications as a function of all suspect identifications, was also low (Clark & Wells, 2008), ranging from 56 to 74%. Note that diagnosticity of 50% would indicate that when a suspect is identified from a lineup, it is no more likely that they are guilty than that they are innocent.

**Table 1:** Proportion of suspect IDs, Filler IDs, and lineup rejections as a function of age and condition

		Short duration Younger child	Short duration Older child	Short duration Adult	Long duration Younger child	Long duration Older child	Long duration Adult
n		245	168	113	241	188	101
Culprit Absent	Filler IDs	0.59 (0.03)	0.57 (0.04)	0.47 (0.05)	0.54 (0.03)	0.55 (0.04)	0.50 (0.05)
	Rejections	0.41 (0.03)	0.43 (0.04)	0.53 (0.05)	0.46 (0.03)	0.45 (0.04)	0.50 (0.05)
Culprit Present	Suspect IDs	0.12 (0.02)	0.17 (0.03)	0.17 (0.04)	0.13 (0.02)	0.16 (0.03)	0.24 (0.04)
	Filler IDs	0.47 (0.03)	0.42 (0.04)	0.47 (0.05)	0.44 (0.03)	0.45 (0.04)	0.36 (0.05)
	Rejections	0.41 (0.03)	0.41 (0.04)	0.36 (0.05)	0.44 (0.03)	0.39 (0.04)	0.41 (0.05)
	diagnosticity	0.56 (0.05)	0.64 (0.04)	0.68 (0.05)	0.59 (0.04)	0.63 (0.04)	0.74 (0.04)
	d’	0.14 (0.11)	0.34 (0.12)	0.46 (0.15)	0.20 (0.11)	0.33 (0.12)	0.66 (0.15)

Standard error shown in parentheses. For d’ standard errors were generated by bootstrapping 10,000 cases by resampling with replacement from the original samples

**Table 2:** Frequency and percentage of lineup decision in culprit present lineups

Lineup version	Culprit	Filler one	Filler two	Filler three	Filler four	Filler five	Filler six	Incorrect rejection	Total
A1	14 (16.09%)	NA	22 (25.29%)	7 (8.05%)	10 (11.49%)	0 (0%)	3 (3.45%)	31 (35.63%)	87
A2	9 (10.47%)	5 (5.81%)	14 (16.28%)	9 (10.47%)	NA	5 (5.81%)	4 (4.65%)	40 (46.51%)	86
A3	17 (19.54%)	9 (10.34%)	NA	11 (12.64%)	1 (1.15%)	4 (4.6%)	3 (3.45%)	42 (48.28%)	87
A4	16 (17.78%)	4 (4.44%)	13 (14.44%)	10 (11.11%)	6 (6.67%)	NA	4 (4.44%)	37 (41.11%)	90
A5	21 (23.86%)	8 (9.09%)	13 (14.77%)	NA	4 (4.55%)	3 (3.41%)	2 (2.27%)	37 (42.05%)	88
A6	15 (16.3%)	10 (10.87%)	10 (10.87%)	5 (5.43%)	2 (2.17%)	2 (2.17%)	NA	48 (52.17%)	92
B1	11 (12.64%)	NA	9 (10.34%)	10 (11.49%)	9 (10.34%)	7 (8.05%)	12 (13.79%)	29 (33.33%)	87
B2	14 (15.56%)	3 (3.33%)	8 (8.89%)	7 (7.78%)	NA	4 (4.44%)	8 (8.89%)	46 (51.11%)	90
B3	9 (10.11%)	3 (3.37%)	NA	12 (13.48%)	14 (15.73%)	6 (6.74%)	10 (11.24%)	35 (39.33%)	89
B4	14 (16.47%)	1 (1.18%)	8 (9.41%)	8 (9.41%)	18 (21.18%)	NA	9 (10.59%)	27 (31.76%)	85
B5	12 (13.95%)	12 (13.95%)	6 (6.98%)	NA	14 (16.28%)	9 (10.47%)	10 (11.63%)	23 (26.74%)	86
B6	10 (11.36%)	8 (9.09%)	6 (6.82%)	7 (7.95%)	15 (17.05%)	7 (7.95%)	NA	35 (39.77%)	88

Under lineup version, Versions A and B refer to the different culprits in the lineups. The numbers refer to the different counterbalances for the culprit-present lineups

**Table 3:** Frequency and percentage of lineup decisions in culprit absent lineups and effective size of lineups by age group

	Age Group					
	5 to 8 years		9 to 13 years		18 years and older	
Lineup Version	A	B	A	B	A	B
Filler One	20 (8.44%)	16 (6.45%)	17 (9.29%)	12 (6.9%)	6 (5.71%)	6 (5.56%)
Filler Two	24 (10.13%)	52 (20.97%)	13 (7.1%)	34 (19.54%)	3 (2.86%)	15 (13.89%)
Filler Three	38 (16.03%)	25 (10.08%)	38 (20.77%)	13 (7.47%)	12 (11.43%)	15 (13.89%)
Filler Four	12 (5.06%)	25 (10.08%)	5 (2.73%)	13 (7.47%)	2 (1.9%)	7 (6.48%)
Filler Five	13 (5.49%)	20 (8.06%)	13 (7.1%)	22 (12.64%)	15 (14.29%)	7 (6.48%)
Filler Six	13 (5.49%)	17 (6.85%)	11 (6.01%)	9 (5.17%)	7 (6.67%)	9 (8.33%)
Correct Rejection	117 (49.37%)	93 (37.5%)	86 (46.99%)	71 (40.8%)	60 (57.14%)	49 (45.37%)
Total	237	248	183	174	105	108
*Tredoux’s E*	4.96	4.90	4.24	4.82	4.34	5.23
*LL*	4.02	3.70	2.50	3.72	3.08	4.67
*UL*	5.46	5.52	5.03	5.36	5.22	5.54

For comparison, in a review of the eyewitness identification literature, Clark et al. (2008) found that the typical (lab-based) eyewitness experiment produced correct rejection rates from culprit absent lineups of around 52% and correct suspect ID rates from culprit present lineups of around 46%. They found that diagnosticity was typically around 77%. Thus, the guilty suspect identification rates in this experiment were quite low compared to the research literature more broadly. Diagnosticity was also considerably lower than is typically observed except for the adult long duration condition. These results indicate that we were successful in generating conditions in which estimator variables were suboptimal.

### Logistic regression

#### Correct rejections from culprit absent lineups

To compare correct rejection accuracy from culprit absent lineups across conditions, we conducted a logistic regression using correct rejection of the lineup as the dependent variable, with duration, age and the interactions as predictor variables. We hypothesized that correct rejections would be higher in adults than older children and higher in older children than younger children. Additionally, we hypothesized that correct rejections would be higher in the long exposure duration condition than the short exposure duration condition. Because there were three age categories, we used two orthogonal contrasts. The first orthogonal contrast compared adult participants to child participants (2 for adults, -1 for young children, -1 for older children). The second orthogonal contrast compared the younger children (− 1) to the older children (1). Results are shown in Table 4. The logistic regression was not statistically significant, χ^2^(5, *N* = 1056) = 5.6, *p* = .347 (Residual deviance = 1448.5 on 1050 degrees of freedom; AIC = 1460.5). The contrast between adults and children was significant (Contrast 1) indicating that adult participants were significantly more likely to reject culprit absent lineups than were child witnesses. There was no significant difference between the two categories of child witnesses. Duration was not a significant predictor, nor was the interaction between duration and either of the contrasts. Additional logistic regression analyses are reported on OSF [https://osf.io/63e2v/].

**Table 4:** Logistic regression showing the relationship between witness age, duration of exposure, and interactions on correct rejection of culprit absent lineups

Predictor	*B*	*SE*	*z*	*p*
Intercept	− 0.173	0.092	− 1.872	.061
Duration (long)	0.037	0.131	0.280	.779
Contrast 1 (Adult = 2, Child = -1)	0.148	0.071	2.080	.038*
Contrast 2 (Adult = 0, Older Child = 1, Younger Child = -1)	0.034	0.101	0.330	.741
Duration X Contrast 1	− 0.090	0.103	− 0.879	.379
Duration X Contrast 2	− 0.053	0.141	− 0.376	.707

*B* = unstandardized regression coefficient; *SE* = standard error. *p < .05

#### Correct identification from culprit present lineups

We performed the same analyses for correct identifications of guilty suspects from culprit present lineups. We hypothesized that hits would be higher in adults than older children and higher in older children than younger children. Additionally, we hypothesized that hits would be higher in the long exposure duration condition than the short exposure duration condition. These results are shown in Table 5. The logistic regression was not significant, χ^2^(5, *N* = 1054) = 8.30,* p* = .139 (Residual deviance = 896.19 on 1048 degrees of freedom; AIC = 908.19). None of the individual predictors were significant.

**Table 5:** Logistic regression showing the relationship between witness age, duration of exposure, and interactions on correct suspect identifications from culprit present lineups

Predictor	*B*	*SE*	*z*	*p*
Intercept	− 1.723	0.127	− 13.613	< .001***
Duration (long)	0.143	0.175	0.816	.415
Contrast 1 (Adult = 2, Child = -1)	0.062	0.096	0.643	.520
Contrast 2 (Adult = 0, Older Child = 1, Younger Child = -1)	0.175	0.142	1.233	.218
Duration X Contrast 1	0.145	0.132	1.098	.272
Duration X Contrast 2	− 0.049	0.198	− 0.249	.803

*B* = unstandardized regression coefficient; *SE* = standard error. *p < .05

### Signal detection analyses

Mickes et al. (2014) recommended comparing discriminability (i.e., d’ values) between conditions to control for response bias. Discriminability is a signal detection measure that takes into account both hits (correct acceptances) and false alarms (incorrect acceptances) to provide an indication of overall memory strength. Correct suspect identifications from culprit present lineups serve as “hits” for this analysis and estimated mistaken suspect identification serve as “false alarms.” Estimated innocent suspect identifications are calculated by taking the proportion of filler IDs from culprit absent lineups and dividing by the number of lineup members (in this case 6) to estimate how often an innocent suspect is likely to be chosen from the culprit absent lineup. Discriminability (i.e., d’) is then calculated by taking the difference between the z score corresponding to hits and the z score corresponding to false alarms.

To maximize power, we compared d’ for adult witnesses to d’ for child witnesses. Using the data from the experiment, we bootstrapped data from 10,000 simulated experiments by resampling from the present experiment with replacement 10,000 times. For each simulated experiment, we calculated d’ for the child witnesses and d’ for the adult witnesses and then calculated the difference between these d’ values (d’_adult_—d’_child_) resulting in 10,000 estimates of the difference between d’ for adults and children. We then calculated the 95% confidence interval based on this bootstrapped data. These results are shown in Table 6. We hypothesized that d’ would be higher for adults than older children and higher for older children than adults. Because the 95% confidence interval of the difference in d’ values did not include zero, we concluded that d’ was significantly higher for adults than for children. We hypothesized that d’ would be higher in the long exposure duration condition than the short exposure duration condition. We performed a similar analysis comparing d’ for the short condition and d’ for the long condition. In this case the bootstrapped 95% confidence interval did include zero, indicating that there was not a significant difference.

**Table 6:** Comparison of d’ values as a function of age and exposure duration, showing upper and lower limit of bootstrapped 95% confidence intervals

	d'	LL	UL
Child Witness	0.242	0.1271	0.3501
Adult Witness	0.56	0.3494	0.7654
Difference	0.32	0.0842	0.5497
Short Duration (6 s)	0.27	0.1258	0.4074
Long Duration (34 s)	0.35	0.2072	0.4800
Difference	0.08	-0.1253	0.2740

### ROC analyses

Some researchers have proposed using partial ROC analyses to evaluate suspect identifications in eyewitness identification experiments (Gronlund et al., 2014; Wixted & Mickes, 2012). The proposal to use ROC analyses has been controversial, with some arguing for their use (Wixted & Mickes, 2015a, 2015b) and others urging caution in their use (Lampinen, 2016; Wells et al., 2015a, 2015b). Lampinen et al., (2019, 2021) demonstrated that for a certain subset of cases, ROC analyses track well with other methods commonly used to analyze eyewitness accuracy. Specifically, Lampinen et al. (2019) noted that when ROC curves are approximately equally truncated along the X-Axis, it is trivially true that the ROC curve with the higher partial area under the curve is associated with the better applied outcome. Moreover, under those circumstances, a range of different proposed measurement techniques will yield the directionally identical applied recommendations (Lampinen et al., 2021). The estimated mistaken suspect ID rate in the present experiment ranges from 7.83% to 9.83%, making the ROC curves approximately equally truncated on Lampinen et al.'s (2019, 2021) typology. For that reason, we analyzed the lineup data using partial area under the curve analyses.

ROC analyses were conducted using pyWitness (Mickes et al., 2023). An ROC analysis plots correct suspect identifications against mistaken suspect identifications at different putative response criterion. As is standard in the field, we use participant provided confidence judgments as proxies for different response criteria. On the ROC curve, the point on the lower left reflects correct suspect identifications and incorrect suspect identifications made with the highest level of confidence. The point above and to the right reflects identifications made with the two top levels of confidence. This continues until all confidence levels are included. Better performance is indicated by higher rates of correct suspect identification for any particular rate of mistaken suspect identifications.

**Fig. 1:**
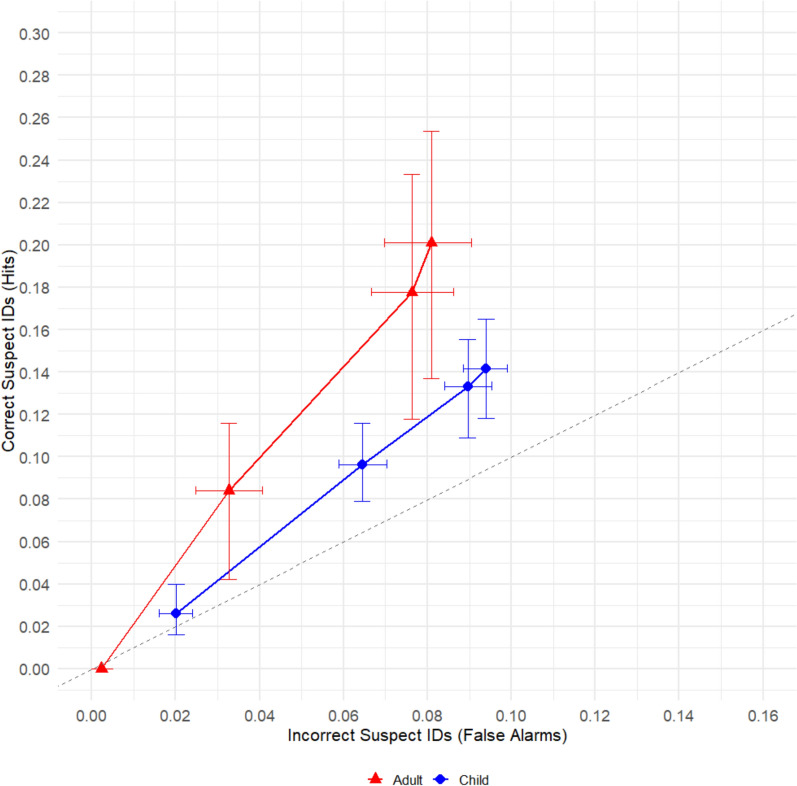
Partial ROC analysis comparing child witnesses and adult witnesses

The ROC curves depicted in Fig. 1 compared performance of adults to children (collapsed across age). We hypothesized that pAUC would be higher for adults than older children and higher for older children than younger children. To compare conditions, we calculated the partial area under each ROC curve out to .084 on the X-Axis, a point shared by both curves. pAUC for adult participants (pAUC = 0.0067 ± 0.0006) was significantly greater than pAUC for child participants (pAUC = 0.0041 ± 0.0008), > *z* = 2.16, *p* = .03.

**Fig. 2:**
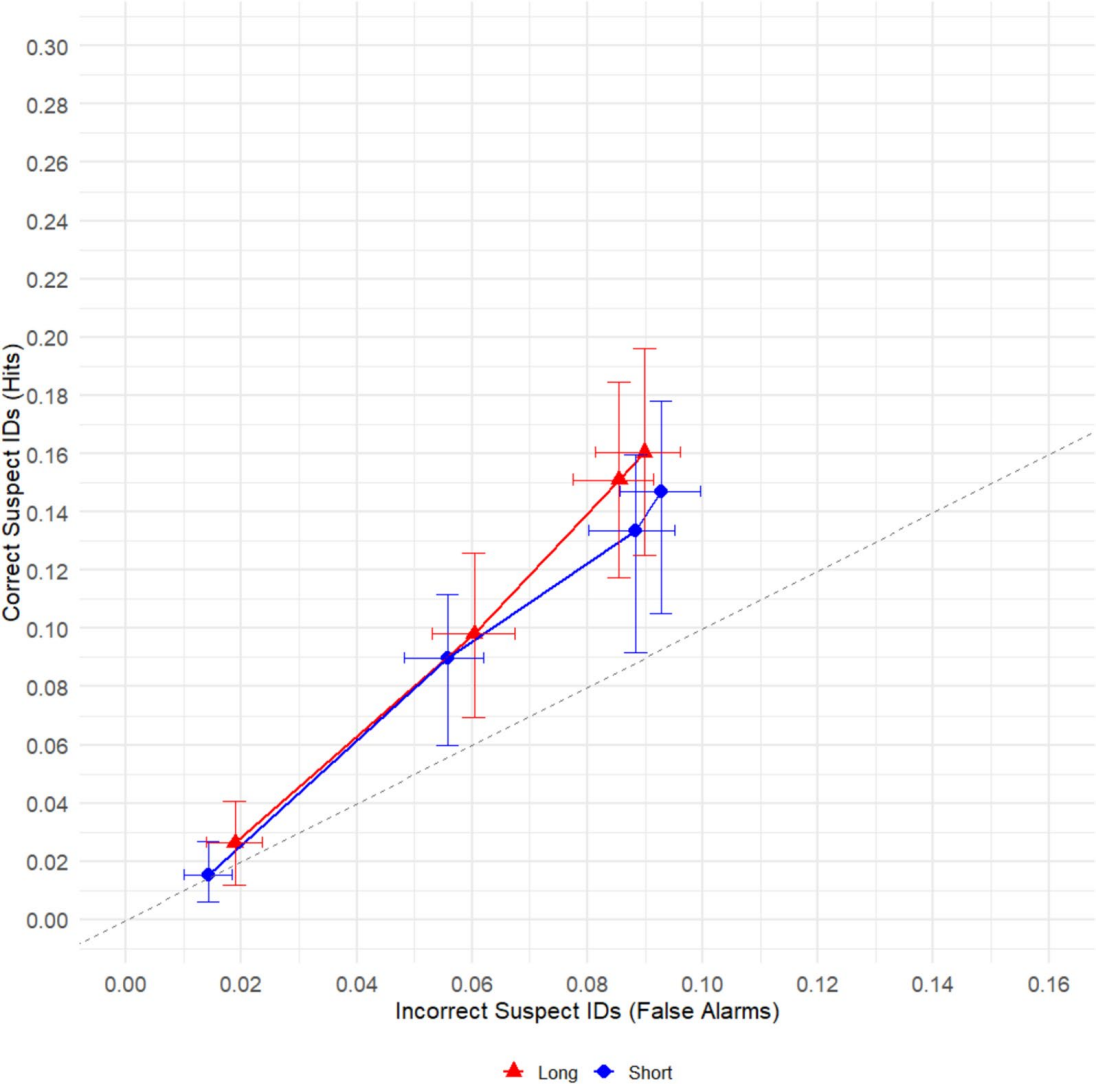
Partial ROC analysis comparing Short vs. Long duration conditions

The ROC curves depicted in Fig. 2 compared performance in the long exposure condition to the short exposure condition. We hypothesized that pAUC would be higher in the long exposure duration condition than the short exposure duration condition. To compare conditions, we calculated the partial area under each ROC curve out to .09 on the X-Axis, a point shared by both curves. The pAUC for the long duration condition (pAUC = : 0.0066 ± 0.0009) did not significantly differ from the pAUC for the short duration condition (pAUC = : 0.0057 ± 0.0009), *z* = 0.70,* p* = .22.

**Fig. 3:**
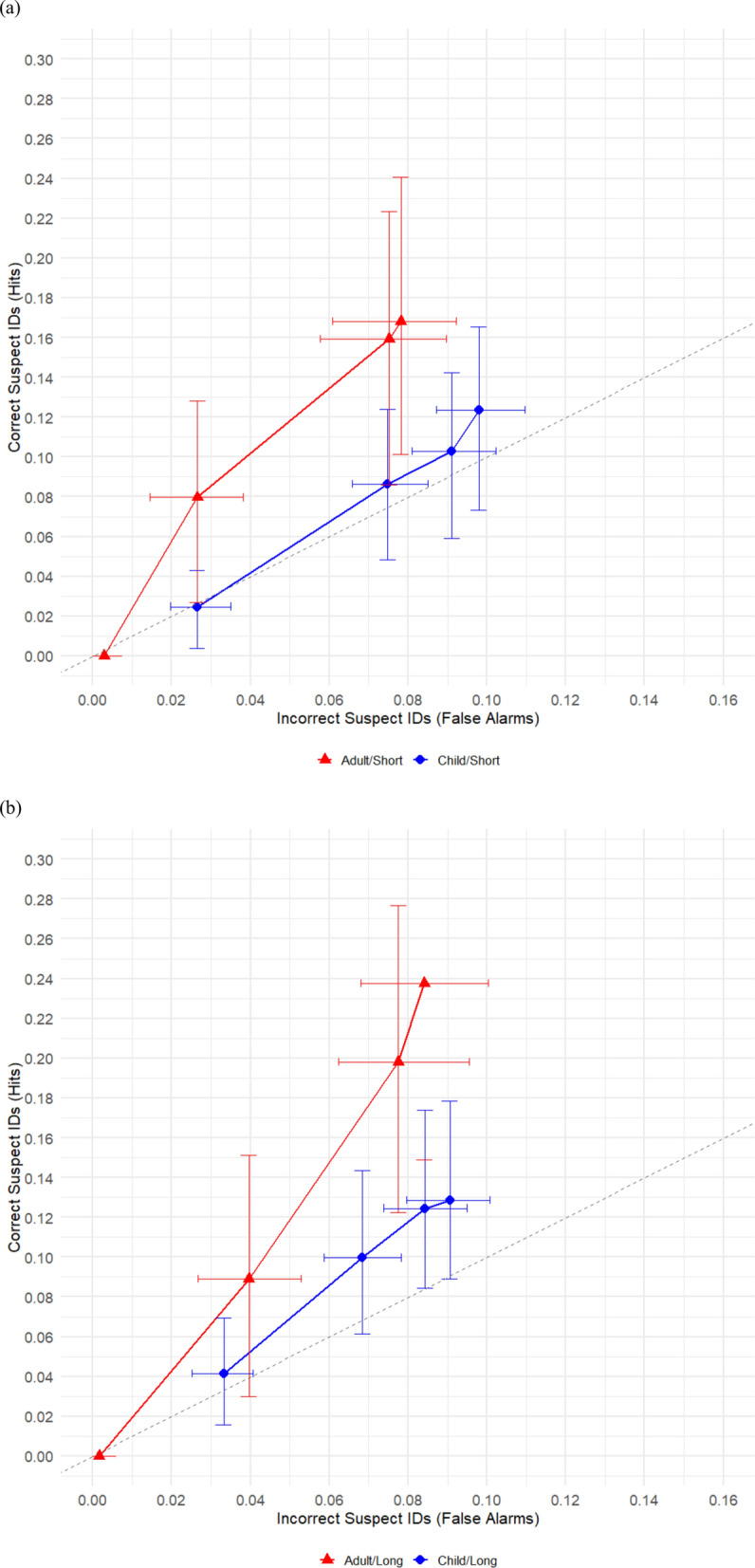
Comparison of children to adults in the short duration condition **a** vs. The long duration condition **b**

We next compared children to adults in the short duration condition and then separately in the long duration condition. Figure 3a shows the ROC curves comparing children with adults specifically in the short duration condition. These comparisons were made out to .078 on the X-Axis, a point shared by both curves. In the short duration condition, pAUC was significantly higher for adult participants (pAUC = 0.0065 ± 0.0008) than for child participants (pAUC = 0.0041 ± 0.0008), *z* = 2.16, *p* = .03. Figure 3b shows the ROC curves comparing children and adults in the long duration condition. These comparisons were made out to .084 on the X-Axis, a point shared by both curves. There was not a significant difference between adult (pAUC = 0.0074 ± 0.0009) and child participants (pAUC = 0.0054 ± 0.0009) for the long duration condition,* z* = 1.57, *p* = .12.

### Eye-tracking attention check

One concern that arises from the poor lineup performance is whether participants were viewing the culprits or looking elsewhere when the critical event occurred. To address this concern, we assessed how long the culprits were in view for a random sample of participants. We used R to randomly select 20% of participants from each age group (i.e., 5 to 8 years, 9 to 13 years, and adults). The second author and two research assistants separately coded 10 participants to ensure agreement on the coding scheme. Then the research assistants coded the video recording of each participant's view for whether the culprit(s) were in view during the critical event. The research assistants coded the time (in seconds) in which both culprits' faces were in view in the video to account for both central and peripheral vision. We started timing when the culprits were as close to the participant as the victim in the video (i.e., standing next to her on the bench).^2^ This value was divided by the total duration the culprits could have been viewed depending on the duration condition. There were also separate instructions on how to code when only one of the culprits were in view, but this happened infrequently (n = 4 for Culprit 1, and n = 9 for Culprit 2).We calculated a two-way, mixed effects intra-class correlation coefficient based on a single rater unit to assess agreement between the research assistants. There was excellent agreement between the two coders, *k* = 0.98, *p* < .001 (Koo & Li, 2016). Since agreement was high, we report one rater’s scores without corrections. Values for each age group and duration condition are displayed in Table 7. We found that on average the culprits were in the participants view the majority of time (*M* = 90–100% for all conditions).

**Table 7:** Average time both culprits were in participants view by age and exposure duration condition

Age group (Years)	Exposure duration condition	Average percent duration in view
5–8 (*n* = 97)	Short	89.96 (23.06)
	Long	93.78 (10.44)
9–13 (*n* = 71)	Short	98.21 (9.45)
	Long	93.64 (14.08)
Adult (*n* = 43)	Short	100 (0.00)
	Long	98.91 (3.66)
Total (*n* = 211)	Short	94.41 (17.63)
	Long	94.86 (11.05)

Numbers in parentheses report the standard deviation of each mean percentage value

### Confidence-accuracy relationship

Prior research has found that even in children, highly confident witnesses are more accurate than less confident witnesses (Winsor et al., 2021). However, the claim of the pristine conditions hypothesis is not just that confidence and accuracy will be related, but that highly confident witnesses will be highly accurate, even when witnessing conditions are poor. In recent research examining the confidence-accuracy relationship in child witnesses, witnessing conditions have tended to be good, as indicated by the relatively high d’ rates that we described in the introduction. We sought to determine if the claim that highly confident witnesses will be highly accurate even in poor viewing conditions holds in children. As noted above, we established conditions in which memory is objectively poor, with estimated d’ ranging from .14 to .66 across conditions. This provides an optimal opportunity for testing the pristine conditions hypothesis.

We evaluated these claims by plotting confidence-accuracy characteristic (CAC) curves (Mickes, 2015). A CAC curve plots estimated suspect identification accuracy at different levels of confidence. Estimated suspect identification accuracy was determined by taking all correct suspect identifications from culprit present lineups and dividing by an estimate of all suspect identifications at that confidence level from both culprit present and absent lineups. Because most eyewitness identification experiments do not designate an innocent suspect, we used a nominal size correction (Clark et al., 2008). Innocent suspect identifications were estimated by taking the total proportion of filler IDs from culprit absent lineups and dividing by the size of the lineup. The logic is that in an experiment, the culprit absent lineup contains a set of innocent individuals (often 6) but in actual police investigations, only one of those six people would be the police suspect. Estimated suspect identification accuracy, at any confidence level, is thus the proportion of guilty suspects identified divided by the proportion of guilty suspects plus the estimated proportion of innocent suspects. A CAC curve plots this estimated suspect identification accuracy at different levels of confidence. In Table 8, we provide the raw number and percentage of lineup decisions by confidence, culprit presence, and age group. High confidence suspect identifications were rare. Among younger children, 3.3% made high confidence suspect identifications on the culprit present lineup and 2.99% made them on the culprit absent lineup (nominally corrected). Among older children, 1.68% made high confidence suspect identifications on the culprit present lineup and less than 1% made them on the culprit absent lineup (nominally corrected). Adults didn’t make any high confidence suspect identifications on the culprit present lineup and less than 1% made them on the culprit absent lineup (nominally corrected).

**Table 8:** Frequency and percentage of lineup decisions by age and confidence in (a) Culprit present lineups and (b) Culprit absent lineups

Age group	Confidence					Total
		1	2	3	4	
*Culprit present lineups*						
5–8 years	Correct ID	6 (11.32%)	10 (11.11%)	29 (14.95%)	16 (10.88%)	61 (12.60%)
	Filler ID	32 (60.38%)	30 (33.33%)	36 (18.56%)	45 (30.61%)	143 (29.55%)
	Rejection	15 (28.30%)	50 (55.56%)	129 (66.49%)	86 (58.5%)	280 (57.85%)
	*n*	53 (10.95%)	90 (18.60%)	194 (40.08%)	147 (30.37%)	484
9–13 years	Correct ID	1 (6.25%)	21 (17.95%)	30 (16.57%)	6 (13.95%)	58 (16.25%)
	Filler ID	8 (50.00%)	34 (29.06%)	30 (16.57%)	13 (30.23%)	85 (23.81%)
	Rejection	7 (43.75%)	62 (52.99%)	121 (66.85%)	24 (55.81%)	214 (59.94%)
	*n*	16 (4.48%)	117 (32.77%)	181 (50.70%)	43 (12.04%)	357
18 + years	Correct ID	5 (23.81%)	20 (19.42%)	18 (21.69%)	0 (0.00%)	43 (20.19%)
	Filler ID	6 (28.57%)	19 (18.45%)	9 (10.84%)	5 (83.33%)	39 (18.31%)
	Rejection	10 (47.62%)	64 (62.14%)	56 (67.47%)	1 (16.67%)	131 (61.50%)
	*n*	21 (9.86%)	103 (48.36%)	83 (38.97%)	62 (2.82%)	213
	Total	90 (8.54%)	310 (29.41%)	458 (43.45%)	196 (18.60%)	1054

*Culprit absent lineups*						
5–8 years	Correct rejection	37 (66.07%)	31 (39.74%)	65 (34.76%)	77 (46.95%)	210 (43.30%)
	Filler ID	19 (33.93%)	47 (60.26%)	122 (65.24%)	87 (53.05%)	275 (56.70%)
	*n*	56 (11.55%)	78 (16.08%)	187 (38.56%)	164 (33.81%)	485
9–13 years	Correct Rejection	15 (83.33%)	49 (37.98%)	74 (42.05%)	19 (55.88%)	157 (43.98%)
	Filler ID	3 (16.67%)	80 (62.02%)	102 (57.95%)	15 (44.12%)	200 (56.02%)
	*n*	18 (5.04%)	129 (36.13%)	176 (49.3%)	34 (9.52%)	357
18 + years	Correct Rejection	8 (57.14%)	45 (44.55%)	50 (56.18%)	6 (66.67%)	109 (51.17%)
	Filler ID	6 (42.86%)	56 (55.45%)	39 (43.82%)	3 (33.33%)	104 (48.83%)
	*n*	14 (6.57%)	101 (47.42%)	89 (41.78%)	9 (4.23%)	213
	Total	88 (8.34%)	308 (29.19%)	452 (42.84%)	207 (19.62%)	1055

Numbers in parentheses in the n per outcome column represent the proportion of response type for all responses in each age group. Numbers in parenthesis for each confidence column represent the proportion of responses at that confidence level and age group out of all of the responses at that confidence level

**Fig. 4:**
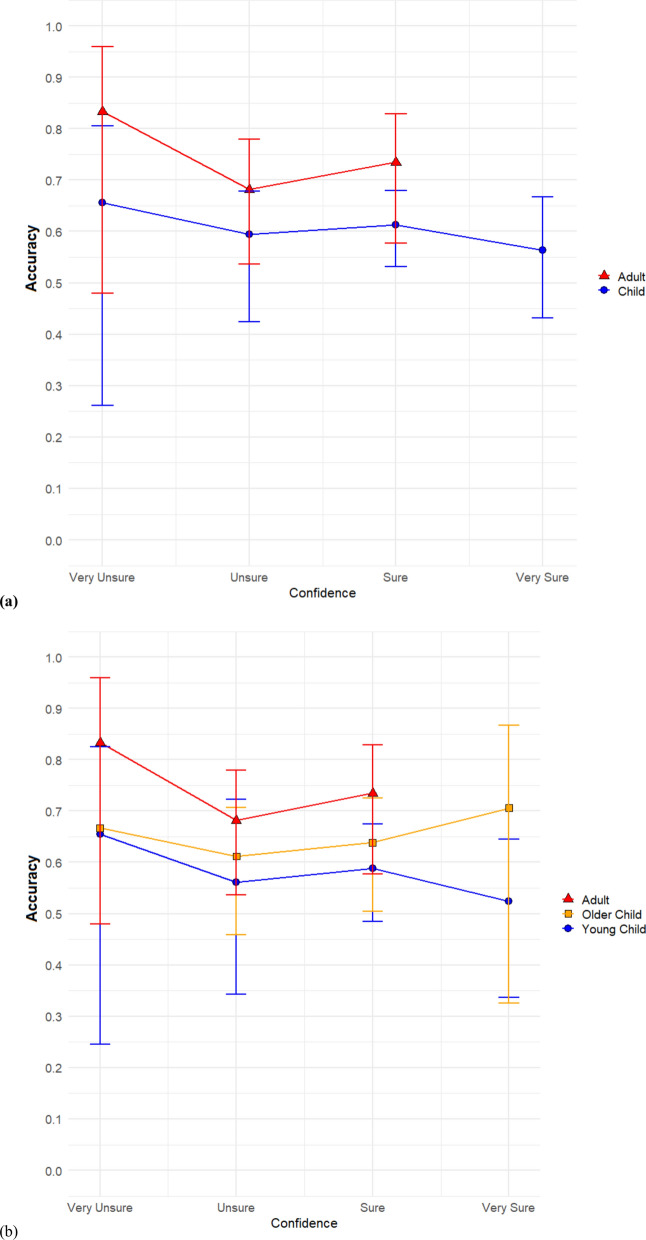
Confidence-accuracy characteristic (CAC) comparisons between child witnesses and adult witnesses. Comparing adults to children collapsed across age **a**. Comparison of adults to younger children and older children **b**

In Fig. 4, we plotted CAC curves comparing child witnesses to adult witnesses. We first compared adults to children, with the child conditions collapsed across age (Fig. 4a). Note that for adult witnesses, there were very few highly confident identifications (confidence = 4) and so the CAC curves are only plotted out to confidence level 3. For children, even at the highest level of confidence, estimated suspect identification accuracy was not high. When child witnesses made an identification with the highest level of confidence, estimated suspect identification accuracy was only about 56%—little better than chance. We next broke the responses up by examining younger children and older children separately (Fig. 4b). For older children the relationship was stronger. Older children who indicated the highest level of confidence, were estimated to be accurate about 70% of the time for their suspect identifications. However, this is still far short of the highly accurate suspect identifications that the pristine conditions hypothesis predicted for highly confident suspect IDs. Additionally, younger children performed slightly above chance.

**Fig. 5:**
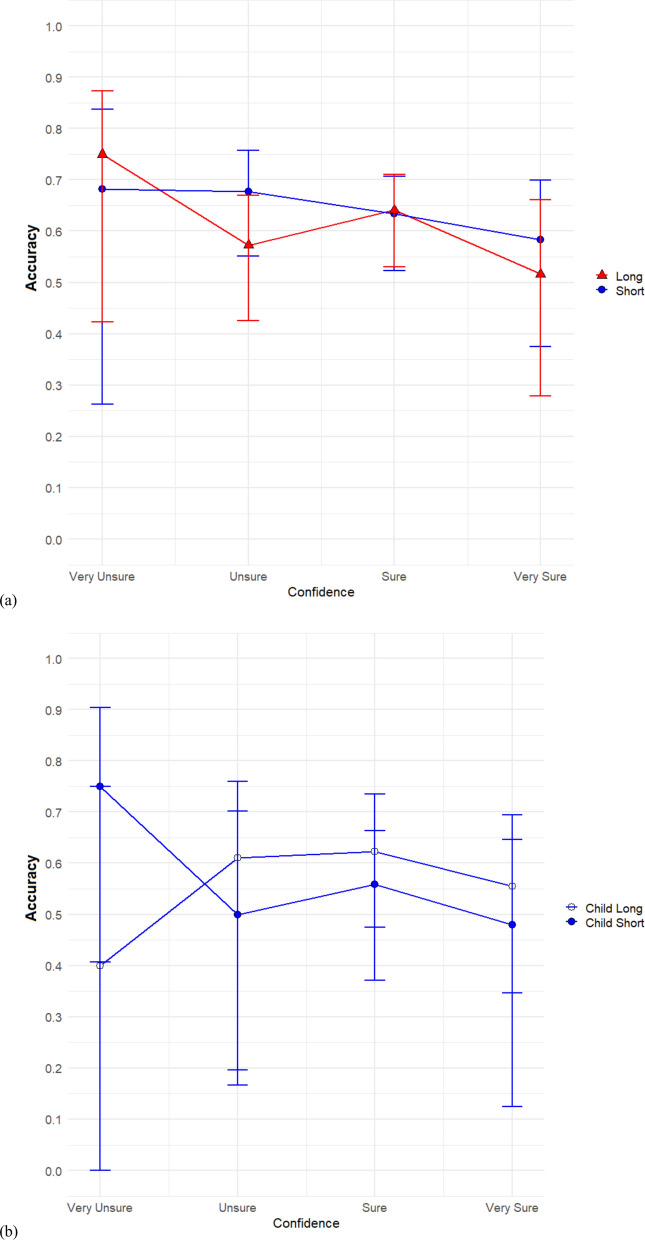

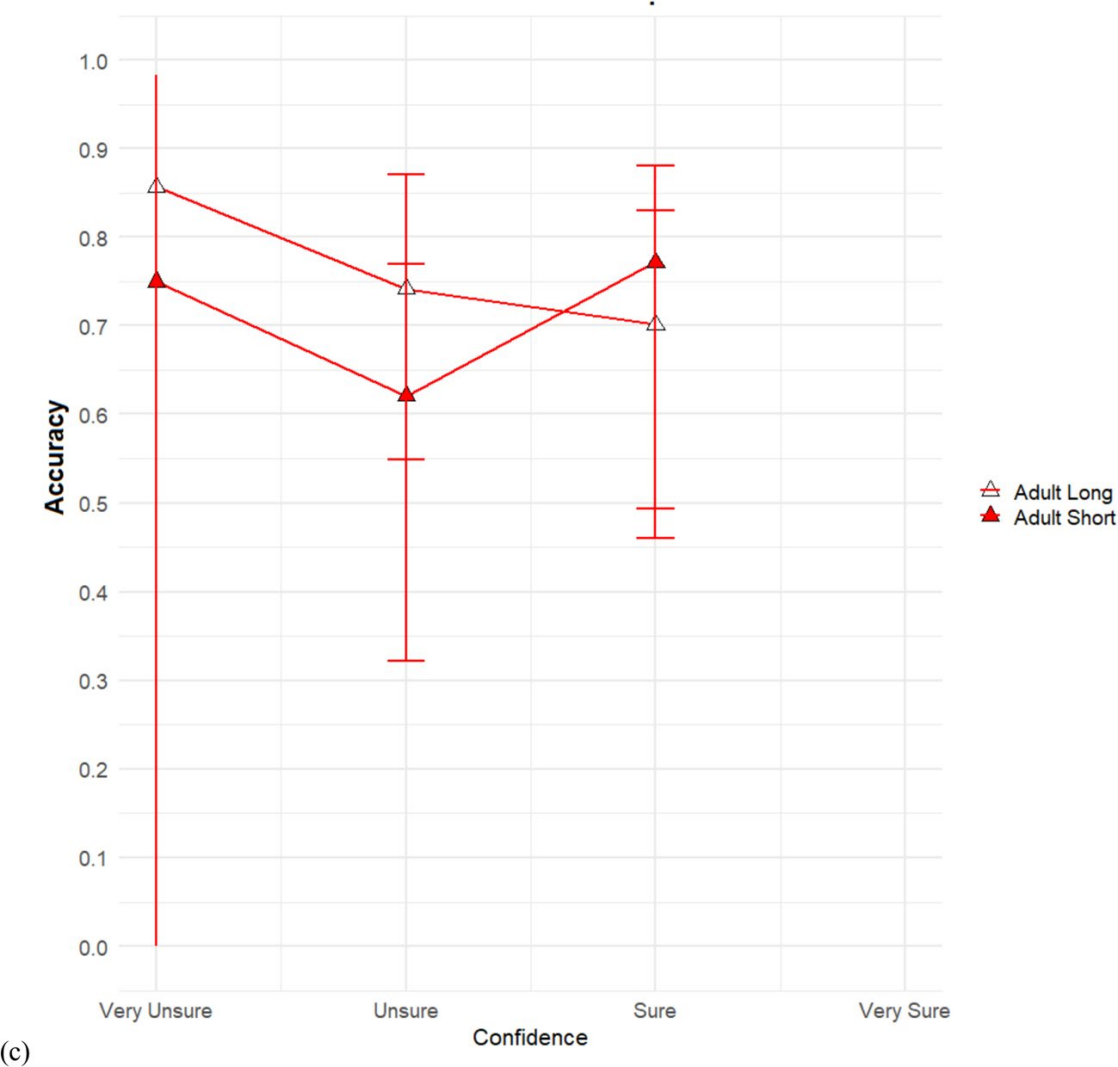
Confidence-accuracy characteristic (CAC) comparisons between short duration and long duration. Comparing short duration to long duration collapsed across all ages **a**. Comparison of short duration to long duration separately for children **b** and adults **c**

Lastly, in Fig. 5, we plotted CAC curves comparing the short duration and long duration condition. We hypothesized that accuracy at high confidence would differ by age only in the brief duration condition with adults having higher accuracy than children. We first do so collapsed across age (Fig. 5a) and then do so broken down by age (Fig. 5b). Note that for both conditions, the estimated suspect identification accuracy, even for highly confident witnesses, was quite low. The estimated suspect identification accuracy for highly confident witnesses in the short duration condition was 52%, while for the long duration condition it was about 58%.

## Discussion

The pristine conditions hypothesis predicts that highly confident witnesses will be highly accurate, even when estimator variable conditions are relatively poor (Wixted & Wells, 2017). We sought to test this hypothesis in children. This is critical because confidence is relied upon as an indicator of eyewitness accuracy in court, yet it is not clear to what extent confidence should be relied upon as an indicator of accuracy especially in child-eyewitnesses. Children and adults viewed a theft of property in a 360-degree, 3D, live-action video in a virtual reality headset. We manipulated exposure duration to create a good and bad estimator variable condition. The theft was committed by two perpetrators, and their faces were visible for either a short or long duration. Participants completed one lineup for each perpetrator, one of which was culprit present and one of which was culprit absent. After each lineup decision, participants provided a confidence judgment. We assessed the confidence-accuracy relationship in children and adults using confidence-accuracy characteristics curves with a particular interest in whether high confidence identifications were remarkably accurate. We were interested in whether high confidence-accuracy varied by age or exposure duration.

Surprisingly, the condition of the estimator variable did not impact the confidence-accuracy relationship. The pristine conditions hypothesis did not hold for child or adult participants under good or bad exposure durations. When children made an identification at the highest level of confidence, estimated suspect identification accuracy was approximately 56%. For adults, high confidence judgments were so rare that accuracy at high confidence could not even be computed. This result occurred even under the ideal condition of a nominal correction, which Fitzgerald et al. (2023) found resulted in an overestimation of accuracy at high confidence due to the underlying assumption of perfect lineup fairness. Older children were more accurate at high confidence than younger children. Exposure duration did not affect lineup identification decisions. We explore several possible explanations in the discussion. In line with the results of Fitzgerald and Price’s (2015) meta-analysis, adults were more likely to correctly reject culprit-absent lineups than children. We found no developmental differences at correctly identifying the guilty suspect in a culprit present lineup. Adults also had better discriminability than children. Overall, memory quality was poor for adults and children (*d*’ = .14 to .66), and this likely explains the lack of effect on exposure duration.

### The pristine conditions hypothesis

Our findings call the utility of the pristine conditions hypothesis into question. Researchers have begun to identify boundary conditions to the pristine conditions hypothesis in adults. Researchers have found that the pristine conditions hypothesis does not hold under certain distances, retention intervals, or multiple poor viewing conditions (Giacona et al., 2021; Lin et al., 2019; Lockamyeir et al., 2020; Nyman et al., 2019, 2025). Additionally, the pristine conditions hypothesis does not hold under certain ages, face recognition abilities, and decision strategies (Colloff et al., 2017; Grabman et al., 2019; Winsor et al., 2021). Critically, the condition of the estimator variables in these studies do not represent extremes at which it would be reasonable to expect the pristine conditions hypothesis to break down. For example, researchers found that high confidence accuracy is not remarkably accurate when there was a retention interval of 10 min (Lin et al., 2019) or when participants were above the age of 30 years old (Colloff et al., 2017). In line with our findings, Nyman et al., (2019, 2025) found that confidence quickly fell so low in suboptimal estimator variable conditions that it was difficult or impossible to calculate the high confidence-accuracy relationship under more realistic conditions than lab studies. Winsor et al. (2021) found that children were highly accurate when highly confident, but our research finds that children are not highly accurate when highly confident. Our results suggest that highly confident child-eyewitnesses are not highly accurate, even when testing conditions are pristine, suggesting that the pristine conditions hypothesis may not generalize to children.

### The pristine conditions hypothesis and memory strength

Recently, Mickes and Wixted (2021) suggested that the high confidence response criterion is driven by overall memory strength, which is adjusted imperfectly as memory strength becomes very weak. According to Mickes and Wixted, “there is undoubtedly some low level of discriminability where the confidence-accuracy relationship will break down." (Mickes & Wixted, 2021, p. 53). Mickes and Wixted do not specify *how* poor memory quality needs to be for this relationship to break down. Importantly, if discriminability is typically poor for actual eyewitnesses or the confidence-accuracy relationship breaks down at a relatively high level of discriminability then the constant likelihood ratio model and assumption that high confidence indicates accuracy is not useful in practice. Notably, the pristine conditions hypothesis (Wixted & Wells, 2017) does not outline such exceptions for poor memory strength—estimator variables should not affect accuracy for high confidence identifications made under pristine conditions. In real cases, we do not necessarily know when or how often memory quality is going to be poor (or how poor it will be). Given that we do not know under which conditions memory strength will be poor, we also do not know when confidence may be indicative of accuracy. This warrants further investigation and consideration before making claims to the court or public that high confidence indicates accuracy.

Semmler et al. (2018) theorize that eyewitnesses adjust their confidence criterion based on the degree of estimator variable suboptimality. That is, people consider estimator variable conditions when making judgments of confidence about their memory strength and do so based on a likelihood ratio (Stretch & Wixted, 1998). One possibility is that children cannot accurately adjust their response criterion to reflect their likely accuracy, perhaps because their confidence calibration and metamemory abilities are not fully developed (Baer & Kidd, 2022; Godfrey et al., 2023). It is worth noting that prior work (e.g., McDonough & Gallo, 2012) highlights that criterion shifts are specifically influenced by beliefs about memorability, not the actual memorability. Similarly, Brewer et al. (2021) showed that answering easy or difficult questions prior to viewing a lineup impacted witness beliefs about their own memory without affecting discriminability. It may be that children do not account for estimator variable conditions because they have limited experience, or because they simply do not hold accurate beliefs about them. It follows that the pristine conditions hypothesis may not hold for child-eyewitnesses *because* metacognitive knowledge is not necessarily accurate. This idea aligns more broadly with recent advances in metacognitive research, which conceptualize confidence as a second-order inferential process that draws on a variety of external cues beyond memory strength, including metamemory beliefs and task-specific features (Fleming & Daw, 2017; Koriat et al., 2008; Rausch et al., 2018; see Shekhar & Rahnev, 2024, for a review). Within the framework of signal detection theory, when discriminability approaches floor levels, it may be theoretically impossible for people to make accurate confidence judgments without relying on these cues (Fleming & Lau, 2014; Maniscalco & Lau, 2012, 2014). Either way, theoretical propositions as to why children cannot use confidence to indicate accuracy when memory quality is poor (even under pristine conditions) have yet to fully explain why this is the case. An updated theory that accounts for metacognitive knowledge is needed to explain when high confidence is likely to indicate accuracy.

### Poor memory strength

Guilty suspect identification rates were low, even in the long duration condition among adults, and diagnosticity was also considerably lower than is typically observed in the literature. It is remarkable that memory strength was so poor given that the stimuli featured good viewing conditions aside from the exposure duration manipulation. In laboratory research, typically strong memory is found even when one estimator variable condition is weak. Eisen et al. (2017) found that eyewitnesses in a field experiment were overconfident and less likely to be correct on showups compared to eyewitnesses in the laboratory. Additionally, Nyman et al., (2019, 2025) studied eyewitness identification under live conditions and found that memory strength was also weak when some estimator variable conditions were poor. While laboratory work has considerably advanced understanding of and policy for eyewitness identification, there may be important witnessing conditions that laboratory work has missed or cannot emulate. Consider for example that people may not have the opportunity to witness a crime in a distraction-free environment. In evaluating the poor memory strength in the current study, we consider the influence of realism including understudied influences on memory strength, multiple culprit crimes, and video resolution as possible explanations.

Virtual reality technology can provide better realism (Newman et al., 2022) than typical 2D laboratory studies. Virtual reality may have provoked additional witnessing variables that are not used or do not occur in 2D video studies, but that likely influence real eyewitness’ memory strength and confidence (e.g., 3D viewing conditions, distractions, angles, immersiveness). For example, participants may have missed witnessing portions of the crime, paid attention to distractions in the environment instead of the crime, or been overwhelmed by the criminal nature of the event they were immersed in despite it being developed to be innocuous and developmentally appropriate. It is not clear what aspect of realism could cause performance to decrease but one apparent hypothesis is that, in 3D, there is more content to view, and the content is more complex because of its realism. This may result in poorer attention to the crime in 3D than for 2D video. Our findings match with that of field studies which find lower accuracy (Nyman et al., 2019, 2025) and more overconfidence than laboratory research (Eisen et al., 2017). Nyman et al. (2020) found higher accuracy on culprit-present lineups for 2D than in Virtual Reality but no difference for culprit-absent lineups. However, work on eyewitness memory for events has found that participants who viewed a crime in virtual reality headsets remembered as much detail about the crime as those viewing it in 2D (Glomb et al., 2024). It is worth investigating whether real eyewitness’ memory is prone to being weaker than in laboratory research.

A second possibility is that memory strength was poor due to the multiple-perpetrator crime. Participants saw two culprits and the victim at encoding. The victim interacted with both culprits. It may be that participants did not pay attention to either culprit given the attention paid to the victim. It may also be that participants' attention was divided between the culprits and thus interfered with their ability to develop a strong memory trace.

Lastly, it is possible that despite using the most cutting edge 360-degree video camera on the market that video resolution for 3D videos embedded in VR programs and headsets may not mimic human vision yet. This may have impacted memory strength. The mock-crime video was shown in 1080p resolution in 3D, which is worse than the same resolution presented in 2D. Additionally, the video quality may have been worse than 1080p in practice because of the impact of routing the video through Unity to display it in the VR headset. In comparison, recent eyewitness studies use 400 × 650 pixels in 2D (e.g., Lockamyeir et al., 2020) while other studies do not report video quality (e.g., Colloff et al., 2021, 2022; Oriet & Fitzgerald, 2018). Therefore, it’s possible that the lower video quality used in the headset created bad witnessing conditions for all participants akin to poor vision or viewing the crime from a distance. As VR technology improves, it will become possible to use better video quality in VR headsets.

### Future directions

In the future, research should aim to examine the impact of estimator variable conditions under realistic conditions when memory strength is stronger on children’s confidence-accuracy. Conducting such research would provide a test of whether the constant likelihood ratio theory is a fit for how children set and adjust their decision criteria for making confidence judgments. In the current research, we intended to provoke such a condition, but memory strength was poor, possibly owing to some of the more realistic conditions that 3D VR affords. Yet, given that our design attempted to mimic real-world conditions, it begs the question of how often strong memory occurs in real-life circumstances. Our findings of poor memory strength provoked many questions about the impact of attention on eyewitness memory strength and confidence, an important topic that researchers have recently drawn more light on (Hyman, 2016; Hyman et al., 2018; Wulff & Hyman, 2022).

## Conclusion

Our research was the first to examine whether the pristine condition hypothesis holds for child-eyewitnesses in the presence of a poor estimator variable. We found that it does not—highly confident participants were not highly accurate. In fact, even when exposure duration was more optimal, participants were not highly accurate when they were highly confident. This research has implications for understanding the utility of eyewitness’ confidence in court.

## Data Availability

Data will be made available publicly upon publication. Materials will be made available upon request.

## Footnotes

1 The choosing rate analyses are available on OSF.

2 This is the same point at which we started measuring the exposure duration for the exposure duration estimates on the short and long exposure duration conditions.
